# Sexual dimorphism in *Caenorhabditis elegans* stress resistance

**DOI:** 10.1371/journal.pone.0272452

**Published:** 2022-08-11

**Authors:** Juan H. Piloto, Michael Rodriguez, Keith P. Choe

**Affiliations:** Department of Biology and Genetics Institute, University of Florida, Gainesville, FL, United States of America; East Carolina University, UNITED STATES

## Abstract

Physiological responses to the environment, disease, and aging vary by sex in many animals, but mechanisms of dimorphism have only recently begun to receive careful attention. The genetic model nematode *Caenorhabditis elegans* has well-defined mechanisms of stress response, aging, and sexual differentiation. *C*. *elegans* has males, but the vast majority of research only uses hermaphrodites. We found that males of the standard N2 laboratory strain were more resistant to hyperosmolarity, heat, and a natural pro-oxidant than hermaphrodites when in mixed-sex groups. Resistance to heat and pro-oxidant were also male-biased in three genetically and geographically diverse *C*. *elegans* strains consistent with a species-wide dimorphism that is not specific to domestication. N2 males were also more resistant to heat and pro-oxidant when keep individually indicating that differences in resistance do not require interactions between worms. We found that males induce canonical stress response genes by similar degrees and in similar tissues as hermaphrodites suggesting the importance of other mechanisms. We find that resistance to heat and pro-oxidant are influenced by the sex differentiation transcription factor TRA-1 suggesting that downstream organ differentiation pathways establish differences in stress resistance. Environmental stress influences survival in natural environments, degenerative disease, and aging. Understanding mechanisms of stress response dimorphism can therefore provide insights into sex-specific population dynamics, disease, and longevity.

## Introduction

Although sex has historically been treated as an inconvenient source of experimental variability, recognition of sex as an important variable in basic animal research is growing [1–3]. Biological sex has broad effects on health, behavior, and interactions with the environment consistent with differences in physiology, biochemistry, and genetics [4–7]. Chronic age-related disease and longevity and are well-established sexually dimorphic traits with broad health and economic implications [8–11]. In humans, females have ~7% longer lifespans than males regardless of geographic region, ethnic background, health system, or wealth [12]; females also live longer in many other species of mammal [13, 14]. Genetic tractability and short lifespans have made *Drosophila* and *C*. *elegans* key models for investigating the genetic basis of aging and longevity [15–17]. Females live longer than males in *Drosophila* [18]. *C*. *elegans* is androdioecious with self-fertilizing hermaphrodites and out-crossing males; hermaphrodites live longer than males when grown in mixed groups as is found in nature, but males live longer than hermaphrodites when keep individually during adulthood [19–21].

Organismal responses to environmental stress have the potential to be sexually dimorphic and influence population dynamics, disease, and aging. Studies in lab rodents and wild mammal populations have demonstrated sex-specific effects of environmental contaminants, harsh weather, and climate change [7, 14, 22, 23]. The sexes of mice, *Drosophila*, and *C*. *elegans* differ in their response to longevity prolonging interventions such as dietary restriction and insulin/IGF-1-like signaling manipulation [21, 24–26]. Cells respond to environmental stress by activating conserved transcription pathways for cytoprotective genes that promote stress resistance [27–30]. Mild exposure to stressors early in life promotes later resistance to extreme stress and increases longevity *via* these pathways [31–33]. Despite their importance to aging, disease, survival, and distributions in nature, few studies have investigated sexual dimorphism in responses to environmental stress.

Stress response mechanisms have been investigated extensively in *C*. *elegans*, but almost exclusively in hermaphrodites [27, 34–38]. Males, which are XO, are generated spontaneously by rare chromosome non-disjunction events or by male mating [39]; non-disjunction rates increase in harsh environments such as high temperature consistent with outcrossing to facilitate adaptation. Hermaphrodites are almost exclusively used in research because they are far more common and easily maintained. It is not known if resistance to common environmental stressors differs between *C*. *elegans* sexes. From an evolutionary perspective, male *C*. *elegans* might be expected to have a greater need for stress resistance because they are more likely to occur in harsh environments and must search for mates. Alternatively, selection for male-specific fitness is likely to be limited because they are rare. Genetic control of sex-differentiation is well-understood in *C*. *elegans* providing a tractable model for mechanisms of dimorphism [40].

We compared stress resistance and stress response gene expression between young adult male and hermaphrodite *C*. *elegans*. In the standard domesticated strain N2, males were more resistant to heat, osmotic, and oxidative stress than hermaphrodites when kept in mixed-sex groups. Heat and oxidative stress resistance were also male-biased in three genetically and geographically diverse natural isolate *C*. *elegans* strains indicating that dimorphism is not unique to domestication. Individual N2 males were also more resistant to heat and oxidative stress than individual hermaphrodites indicating that differences in resistance are not dependent on interactions between worms. Comparisons of stress response gene expression under basal conditions was complicated by gross differences in anatomy and tissue-specific gene expression. Males induced canonical heat and oxidative stress-response genes by similar levels and in the same major tissues as hermaphrodites suggesting that other mechanisms drive differences in stress resistance. Lastly, resistance to heat and oxidative stress were influenced by sex determination master regulator TRA-1 suggesting that downstream developmental mechanisms establish differences in stress resistance.

## Materials and methods

### Worm strain and maintenance

The strains used were: wild-type N2 Bristol, AB2, CB4856, CB4853, CL2166 *dvIs19*[*gst-4p*::*GFP*], VP604 *kbIs24*[*gpdh-1p*::*DsRed2;myo-2p*::*GFP;unc-119 rescue*], QV65 *gpIs1*[*hsp-16*.*2p*::*GFP*];*vsls33*[*dop-3p*::*DsRed2*], and CB2590 *tra-1(e1099)/dpy-18(e1096) III*. All worms were maintained at 20°C using standard methods [41]. To maintain CB2590, individual L4 wild-type hermaphrodites were isolated and checked for segregation of Dpy and pseudomale phenotypes in offspring.

Mixed-sex populations of wild type worms were maintained by picking a high ratio of males to hermaphrodites every few generations. Gravid adults from these mating populations were bleached and the following synchronized generation was used for survival and gene expression experiments. Males and hermaphrodites were grown together on the same agar plates during larval development.

### Physiological assays

Some agar plates were supplemented with either 400–425 mM NaCl or 175 μM juglone. Synchronized males and hermaphrodites were grown together until the first day of adulthood and then transferred together by chunking and immediate removal of the chuck leaving worms on the surface of agar containing high NaCl or juglone. Survival on high salt was scored at 24 h as described previously [42, 43]. Juglone has a short half-life causing high variability between batches of agar [44, 45]. Therefore, survival of juglone was scored at 12 and 24 h and the earliest time point when at least 50% of worms in one sex were dead was used for analysis [46]. For heat stress, standard agar plates with first day adult worms were wrapped in parafilm, floated on a water bath at 35°C for 8 h, transferred to 20°C, and scored for survival starting 12 h after recovery as described previously [47]. Worms on agar were counted dead if they did not respond to gentle touching with a wire or hair pick. Each stressor plate containing a population of both sexes served as a replicate for survival analysis; each trial was defined as a separate batch of synchronized worms.

For testing heat shock and juglone survival of individual worms, single L4 male and hermaphrodite worms from synchronized mixed-sex populations were picked to each well of a 96 well plate; each well contained OP50 bacteria at a final OD of 1.8–2.0, 100 μg/ml streptomycin, and 50 μg/ml carbenicillin in a total of 100 μl of liquid NGM buffer. When worms reached the young adult stage, they were exposed to stressors. For heat, the edges of plates were sealed with parafilm and they were floated on a 35°C water bath for 8 h, transferred to 20°C, and scored for survival starting 12 h after recovery. Juglone was added to a final concentration of 175 μM. Worms in liquid were counted dead if they were immobile, had a rigid posture, and did not respond to tapping of the plate.

### qRT-PCR

Quantitative RT-PCR assays were performed as described previously [48] with the following modifications. Each sex was picked at the young adult stage before containing embryos and frozen in separate tubes. After lysis, genomic DNA was degraded using dsDNAse according to manufacturer’s protocol (Thermo Fisher product EN007). Stress response mRNA levels were normalized to *cdc-42* and *Y45F10D*.*4* and averaged for each sample. PCR products were verified with single melt curves and no template controls. Primer sequences are in S1 Table. To measure stress-induced gene expression, worms were treated with sub-lethal 250 mM NaCl, 87.5 μM juglone, or exposed to a 35°C water bath for 24 h, 1 h, or 15 minutes, respectively.

### Fluorescence analyses

Worms were mounted on agarose pads with 5 mM levamisole and imaged using an Olympus BX60 microscope with a Zeiss AxioCam MRm camera fitted with either GFP or RFP filters. Exposure settings were consistent within each strain regardless of condition or sex. Color was added using PowerPoint or ImageJ Version 1.53c in cases where two colors were merged; adjustments to contrast and brightness were made evenly to whole images and identically within each strain for fluorescence. Images presented are representative of at least 10 worms.

### Statistical analyses

Statistical significance for qRT-PCR experiments was determined with two-tailed unpaired t-tests with Benjamini-Hochberg false-discovery rate adjustments when multiple genes were measured. Statistical significance for NaCl and heat survival assays on agar was determined with two-tailed unpaired Student’s t-tests. Paired Student’s t-tests were used for juglone survival to control for batch effects caused by low stability of juglone [44]. Statistical significance for individual heat shock survival was determined with Log-rank tests using the OASIS online statistic tool [49].

## Results and conclusions

### N2 males are more resistant to environmental stress than hermaphrodites in groups

**Fig 1:**
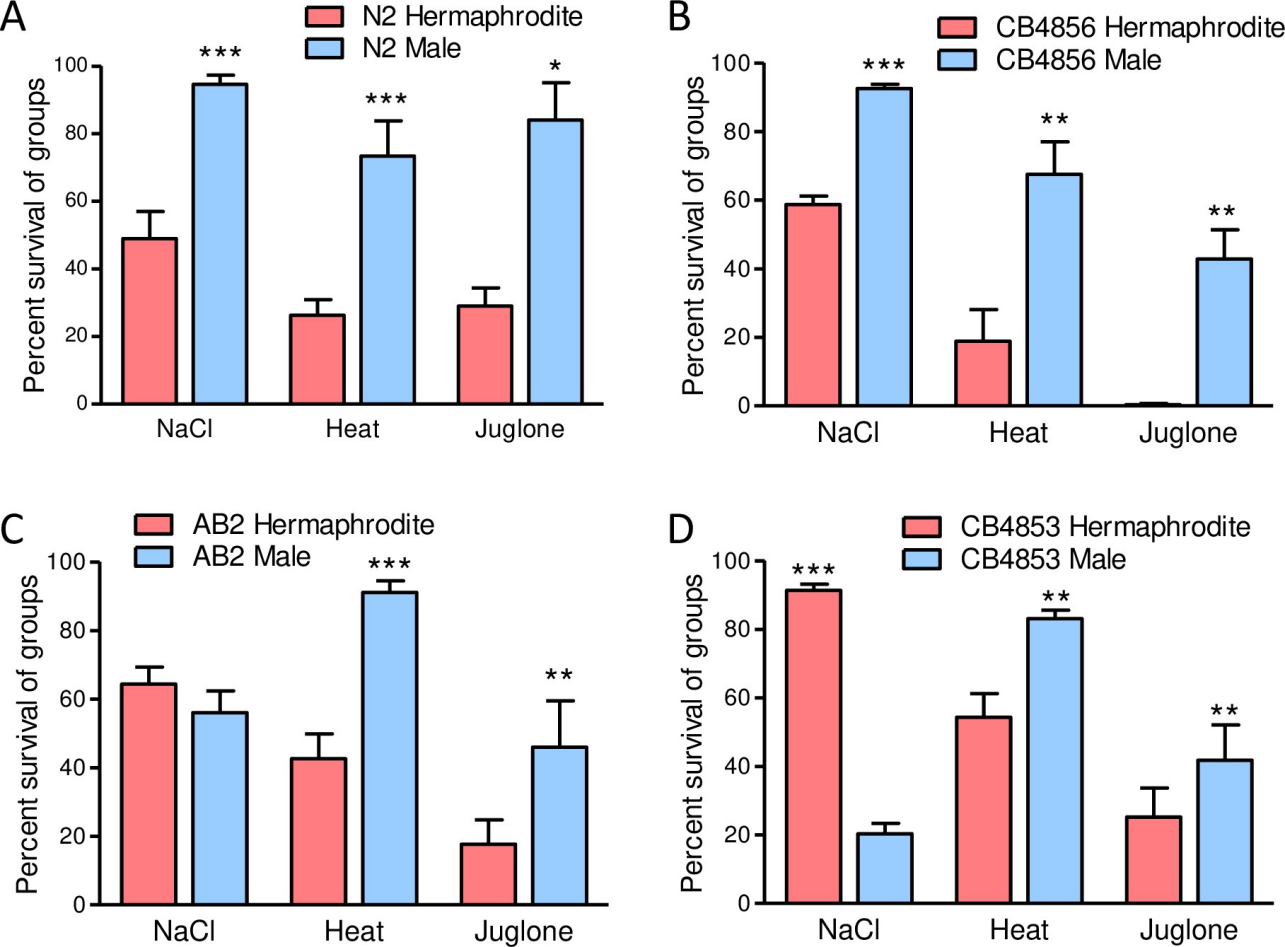
Males are more resistant to osmotic stress, heat shock, and juglone than hermaphrodites in groups. (A-D) Male and hermaphrodite worms were growth on standard 51 mM NaCl agar until the young adult stage and then exposed together in groups to 425 mM NaCl, 35°C for 8 h, or 175 μM juglone. Survival was scored after 24 h for 425 mM NaCl, 72 h of recovery for heat shock, and 12–24 h for juglone. Values are mean ± standard error. *N* = 4–12 replicate populations of 17–920 worms from 2–3 trials. *P > 0.01,**P < 0.01, and ***P < 0.001 versus hermaphrodites.

We first tested if *C*. *elegans* sexes differ in resistance to stress in the common laboratory strain N2. We used three environmental stressors that are used widely in the literature and are found in natural environments of free-living nematodes: hyperosmolarity, heat, and juglone. Hyperosmolarity causes loss of cell volume and protein aggregation [42, 50–52]. Juglone is a natural allelochemical and pro-oxidant produced by plants of the juglandaceae walnut family to inhibit growth of other organisms in surrounding soil [53]. High temperature causes protein misfolding [54]. Changes in osmolarity and temperature are likely common for *C*. *elegans* in rotting surface vegetation with changes in time of day, sun exposure, rain, humidity, and plant tissue osmolarity. Exposure to juglone and similar nathoquinones is also likely, because juglandaceae has a broad distribution [55]. As shown in Fig 1A, N2 males were more resistant to all three stressors than hermaphrodites when together in mixed-sex populations.

### Males of natural *C*. *elegans* isolates are more resistant to heat and juglone than hermaphrodites

We next tested if the male-biased stress resistance we observed in Fig 1A is representative of the species or specific to N2, which is a domesticated strain derived from an isolate in Bristol, England (77). We selected three natural isolates that are genetically and geographically divergent. CB4856 is from Hawaii and is one of the most genetically divergent from N2 [56]. AB2 is from Adelaide, Australia, and CB4853 from Altadena, California [57, 58]. As shown in Fig 1B–1D, resistance to heat and juglone was male-biased in all three natural isolates; together with data for N2 (Fig 1A), these results are consistent with a species-wide male-bias in resistance to acute heat shock and oxidative stress when worms are in mixed-sex groups as found in nature. Alternatively, sex-bias in high salt resistance varied greatly by strain (Fig 1A–1D); it was male-biased in N2 and CB4856, there was no bias in AB2, and it was hermaphrodite-biased in CB4853.

### Individual N2 males are more resistant to heat and juglone than hermaphrodites

**Fig 2:**
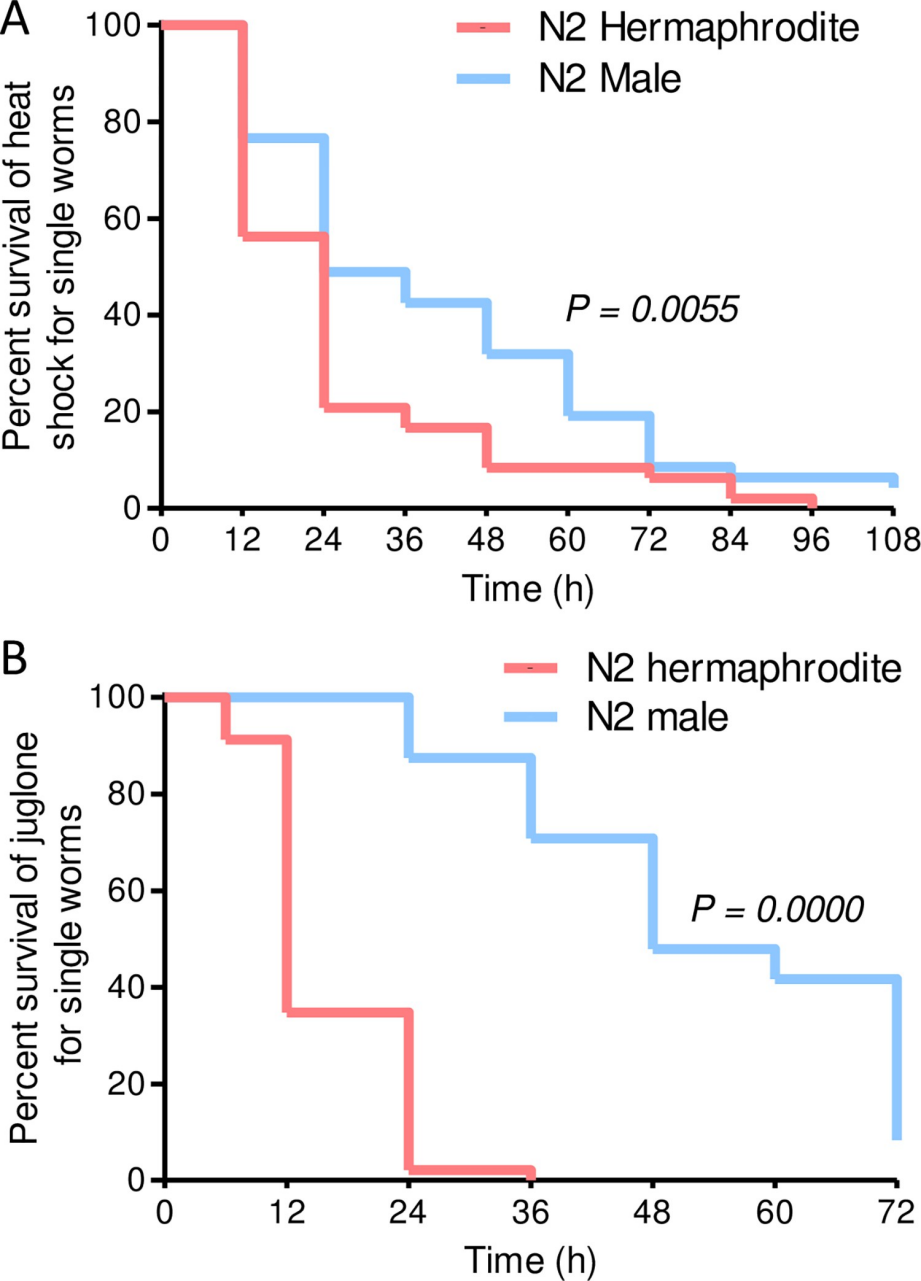
Males are more resistant to osmotic stress, heat shock, and juglone than hermaphrodites as individuals. Survival curves for single trials heat shock (A) or juglone (B); details for all trials are in S1 Fig. Male and hermaphrodite worms were growth together on standard 51 mM NaCl agar until the L4 larval stage; one L4 worm was picked to each well of a 96 well plate containing OP50 bacteria in liquid NGM buffer. On the first day of adulthood, worms were exposed to 35°C for 8 h or 175 μM juglone was added. Survival was scored for each worm. *N* = 47–49 worms of each sex.

Prior studies demonstrated that sex-bias in longevity is influenced by pheromones and physical interactions between worms with males having a longer lifespan than hermaphrodites when kept individually during adulthood but a shorter lifespan than hermaphrodites when kept in groups [19–21]. For comparison to these prior results, we also tested survival of N2 males and hermaphrodites that were picked individually to wells of a microplate at the L4 larval stage and then exposed to heat shock or juglone on the first day of adulthood. As shown in Fig 2A, 2B and S1 Fig, males were more resistant to heat shock and juglone when isolated as individuals. The dimorphism was particularly strong for juglone with males having a mean survival time more than threefold longer than hermaphrodites (Fig 2 and S1 Fig).

### Basal stress response gene expression

Organisms respond to stress, in part, by activating expression of genes encoding proteins that repair damage or diminish the stressor. For example, protein homeostasis mechanisms and organic osmolyte synthesis and transport promote survival of hypertonicity [42, 50, 51, 59, 60]. Detoxification and anti-oxidation genes regulated by transcription factors SKN-1 and DAF-16 promote survival of juglone [46, 61, 62]. Small cytosolic chaperones regulated by HSF-1 and DAF-16 promote survival of high temperature [54, 63]. We first used qRT-PCR to compare expression of stress response genes under basal conditions. We measured mRNA levels for well-characterized genes representative of core hyperosmotic (*gpdh-1*), endoplasmic reticulum (ER) unfolded protein (*hsp-4*), SKN-1 dependent detoxification (*gst-4*), HSF-1 dependent cytosolic heat shock (*hsp-16*.*2*), mitochondrial unfolded protein (*hsp-6*), metal (*mtl-2)*, and RNA homeostasis (*numr-1/2*) stress responses [46, 59, 64–67]. Expression was normalized to two housekeeping genes (*cdc-42* and *Y45F10D*.*4*).

N2 males had 5.4, 4.5, 2.6, 3.7, 2.0, and 4.5-fold greater relative mRNA levels for core osmotic, ER stress, detoxification, heat shock, metal response, and RNA homeostasis genes than hermaphrodites, respectively (S2A Fig); mitochondria and innate immune response genes were not statistically different. Although these results suggest higher basal expression of some stress response genes in males, interpretation of these differences is complicated by sex-specific organs (e.g., spermatheca, uterus, vulva, seminal vesicle, and vas deferens) and potential differences in abundance of tissues enriched in stress-response mRNAs such as epidermis, intestine, and muscle [43, 68–70]. We also compared expression of tissue-specific mRNAs that are not known to be regulated by stress: *ctsa-1*.*2* (intestine), *elt-3* (intestine), *elt-7* (intestine), *vha-6* (intestine), *lon-1* (epidermis and intestine), *nhr-23* (epidermis), *col-19* (epidermis), and *mlc-1* (muscle). Expression of *ctsa-1*.*2*, *nhr-23*, and *mlc-1* were 2.4–2.7-fold greater and *elt-3* and *lon-1* were 2-fold lower in males than hermaphrodites consistent with general sex-biases in tissue abundance and/or gene expression that account for at least 2-3-fold differences (S2B Fig). Expression of *gpdh-1*, *hsp-4*, and *numr-1/2* were slightly more male-biased than a 3-fold threshold (S2A Fig), but highly divergent anatomy makes interpreting the biological importance of these differences difficult.

### Males induce stress response genes similar to hermaphrodites

**Fig 3:**
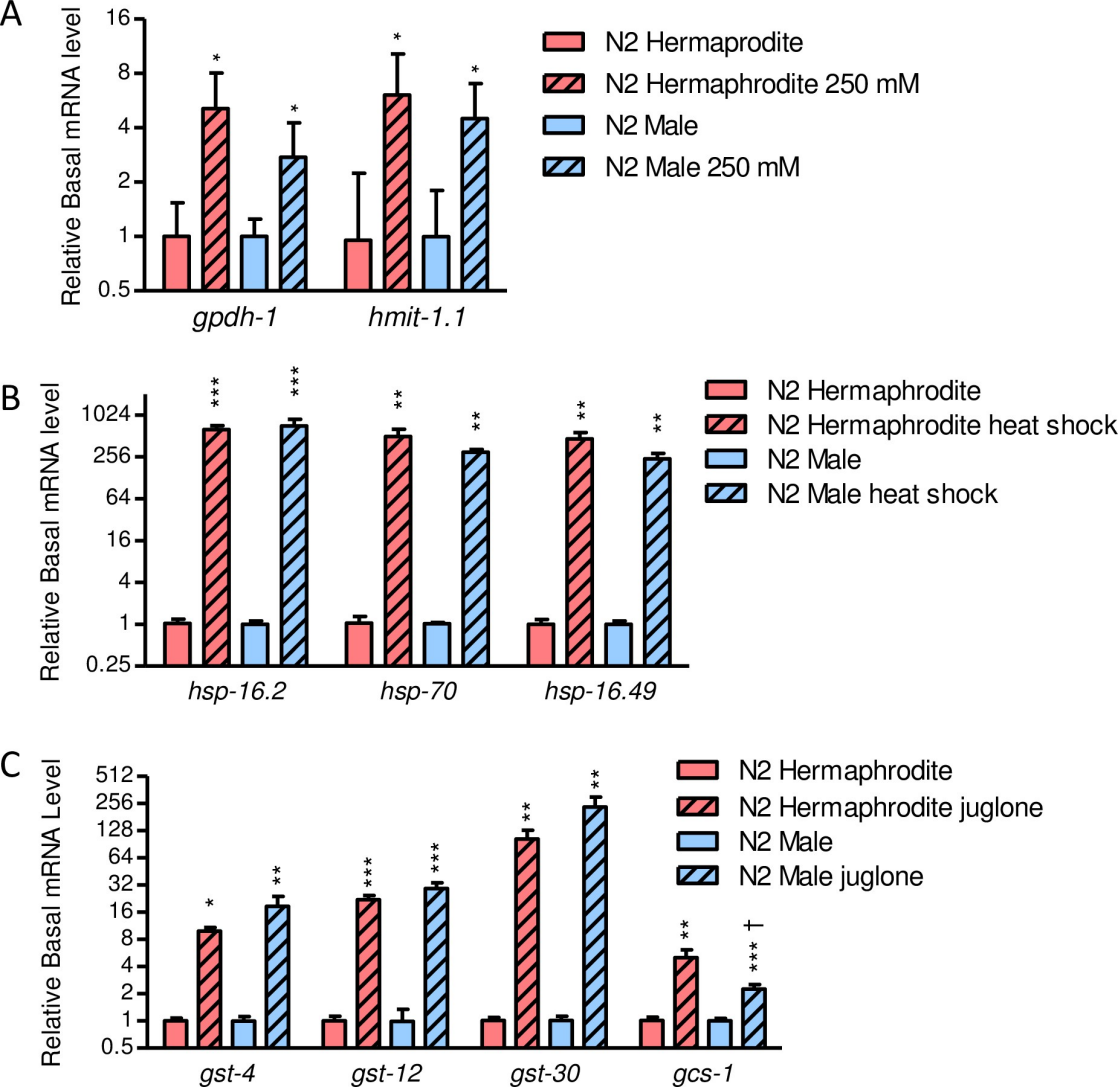
Stress response gene induction in N2 males and hermaphrodites. Expression of stress response genes with and without exposure to (A) osmotic (250 mM NaCl for 24 h), (B) heat shock (35°C for 15 min), or (C) oxidative stressors (87.5 μM juglone for 1 h). Values are mean plus standard error. *N* = 4–10 replicate populations of 3–5 worms. *P < 0.05, **P < 0.01, and ***P < 0.001 versus control of the same sex; ^†^*P* < 0.05 versus hermaphrodites of the same condition.

Gene induction by stress is well documented in hermaphrodites but not in males. We next compared the ability of males and hermaphrodites to induce stress responses. Both sexes of N2 were exposed to sublethal doses of stress (250 mM NaCl, a 35°C water bath, or 87.5 μM juglone for 24 h, 15 minutes, and 1 h, respectively). Males induced hyperosmotic (*gpdh-1* and *hmit-1*.*1*), heat shock (*hsp-16*.*2*, *70*, and *16*.*49*), and detoxification (*gst-4*, *gst-12*, and *gst-30)* genes by levels comparable to hermaphrodites (Fig 3). Induction of only glutathione synthesis gene *gcs-1* was significantly different between sexes with a reduced induction in males (2.3-fold versus 5.0-fold in hermaphrodites, Fig 3C). Therefore, males strongly induce osmotic, heat shock, and detoxification genes, but not more than hermaphrodites.

**Fig 4:**
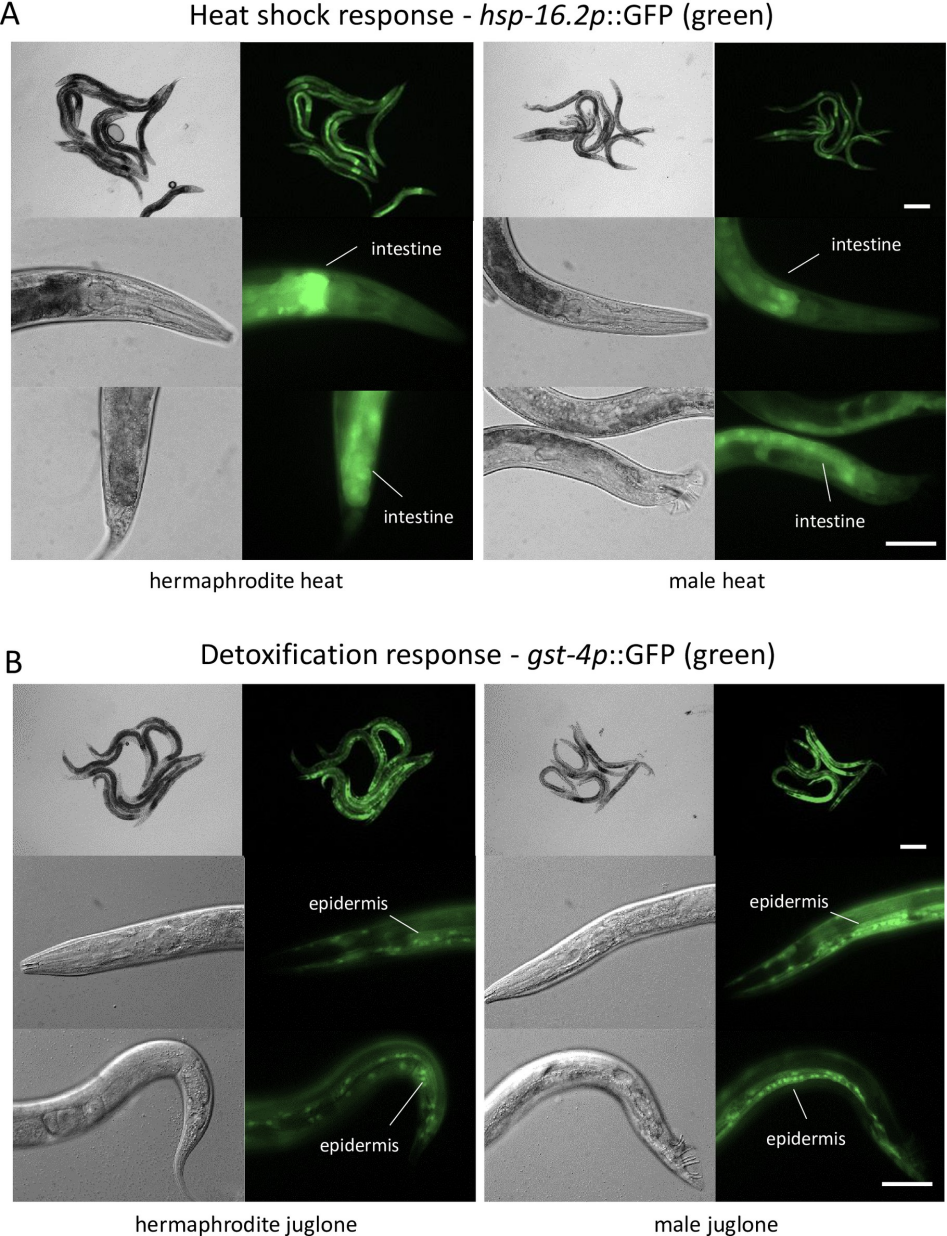
Males induce *hsp-16*.*2* and *gst-4* in the same tissues as hermaphrodites. Paired bright-field and fluorescence micrographs of *hsp-16*.*2p*::GFP (A) and *gst-4p*::GFP (B) expressing worms after heat shock (12 h after 35°C for 1 h) or juglone exposure (12 h after 87.5 μM juglone for 1 h). Images of the same magnification and strain were taken with the same exposure settings. Scale bars are 200 or 50 μm at low and high magnification, respectively. Images are representative of at least 10 worms.

Given difficulties interpreting basal mRNA differences and lack of correlation between stress response induction levels and male-biased stress resistance, we next used fluorescent transcriptional reporters for canonical osmotic, heat shock, and detoxification stress response genes (*gpdh-1p*::*dsRed2*, *hsp-16*.*2p*::*GFP*, *and gst-4p*::*GFP*, respectively) [46, 71, 72] to determine if there were any major differences in distributions that may correlate with stress resistance. As shown in S3A and S4 Figs, fluorescence for these reporters was not clearly visible above background in either sex under basal conditions. Fluorescence for all three reporters was strongly induced in both sexes by their respective stressors; *gpdh-1p*::*dsRed2* was induced in the intestine by high salt (S3B Fig), *hsp-16*.*2p*::*GFP* was induced in the intestine by heat shock (Fig 4A), and *gst-4p*::*GFP* was induced in the epidermis by juglone (Fig 4B). Therefore, these canonical osmotic, heat shock, and detoxification stress response genes are induced in the same major tissues in both sexes.

### Sex-determination factor TRA-1 influences stress resistance

We next tested if the central sex-determination pathway of *C*. *elegans* influences stress resistance. Development of many sexually dimorphic characteristics is determined by a cascade of signaling proteins terminating with transcription master regulator TRA-1 in *C*. *elegans* [40, 73]. Sex chromosome dosage determines if the pathway regulating TRA-1 is active; in XX worms, TRA-1 is de-repressed allowing it to repress male development; in XO worms, TRA-1 is repressed and male organs development. XX worms homozygous for a strong loss of function *tra-1(e1099)* allele develop into phenotypic ‘pseudomales’. XX *tra-1* pseudomales have tails and non-gonadal tissues that are indistinguishable from XO wild type males; compared to wild type males, *tra-1* pseudomale gonads are smaller and mating success is reduced [74].

**Fig 5:**
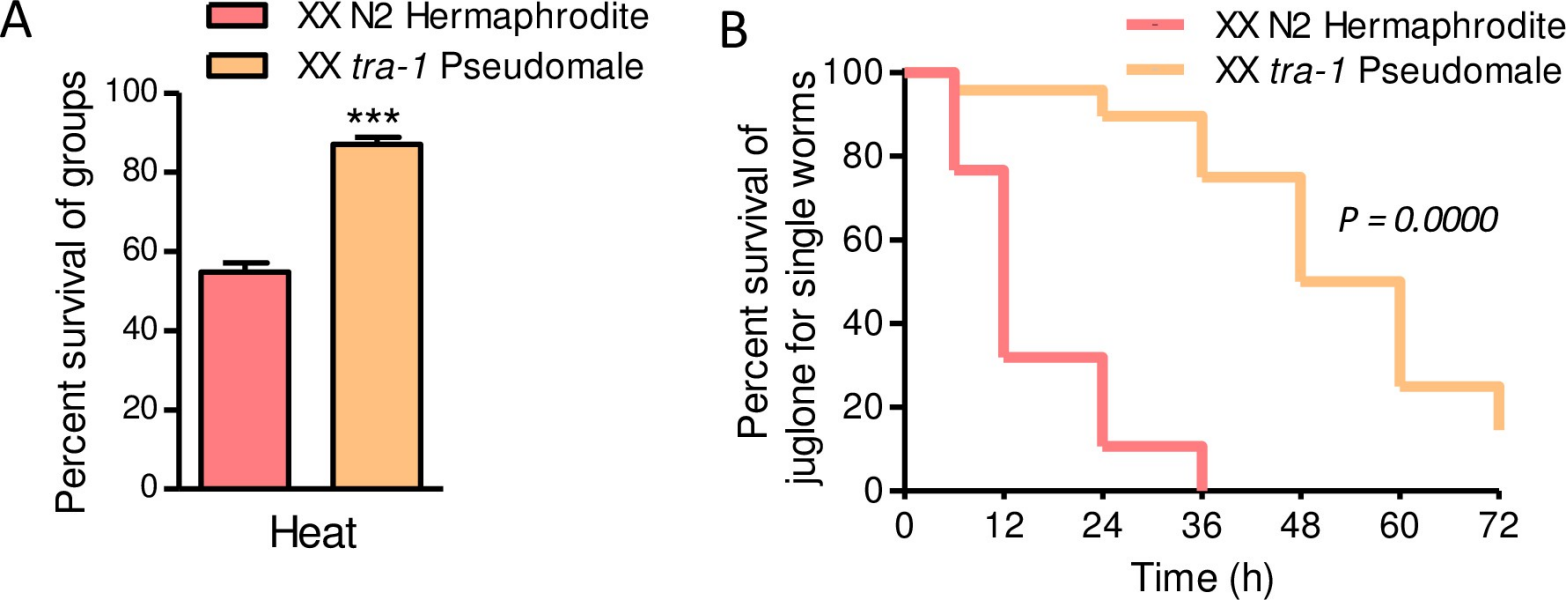
XX *tra-1* pseudomales are more resistant to heat and juglone than hermaphrodites. (A) Heat shock survival for N2 wild type hermaphrodites and homozygote *tra-1(e1099)* pseudomales; heat shock was 35°C for 8 h and then 72 h of recovery. Values are mean ± standard error. N = 4–6 replicate populations of 27 to 359 worms from 2 trials. (B) Survival curves for *tra-1* pseudomales and N2 hermaphrodites in 175 μM juglone; details for all trials are in S1 Fig. Assay conditions were identical to Fig 2B.

We tested resistance to heat shock and juglone, which were consistently male-biased in wild type worms (Figs 1 and 2, and S1 Fig). As shown in Fig 5A, XX *tra-1* ‘pseudomales’ were more resistant to heat shock than wild type hermaphrodites in groups; heterozygote hermaphrodite survival was indistinguishable from wild type hermaphrodites (51.5±5.9%, N = 8). As shown in Fig 5B and S1 Fig, XX *tra-1* ‘pseudomales’ were more resistant to juglone than wild type hermaphrodites when isolated as individuals; in a trial that also included N2 males, there was no difference between wild type males and *tra-1* pseudomales (S1 Fig). These results suggest that sexual characteristics established downstream from active TRA-1 in wild type XX hermaphrodites reduce resistance to heat shock and juglone relative to males, which have low TRA-1 activity.

We also measured stress response gene expression under basal conditions. As shown in S5 Fig, XX *tra-1* ‘pseudomales’ had 2.5–3.0-fold greater expression of core osmotic, detoxification, heat shock, innate immune, metal response, and RNA homeostasis genes than wild type hermaphrodites. However, similar to wild type males, intestine-enriched mRNAs *csta-1*.*2* and *elt-7* were also expressed higher in *tra-1* ‘pseudomales’ by similar degrees making interpretation of these differences difficult.

## Discussion

### *C*. *elegans* males are more resistant to heat shock and juglone than hermaphrodites

Results in Fig 1 demonstrate that *C*. *elegans* males are more resistant to acute heat shock and oxidative stress than hermaphrodites when living together as they do in nature. The four strains that we tested are genetically and geographically diverse consistent with this male-bias being species-wide and not a byproduct of domestication. Greater resistance to heat shock and toxins would be expected to give males an advantage in harsh conditions that increase their occurrence due to X chromosome non-disjunction.

Greater resistance to stress is often correlated with slower aging and longer lifespan [75–77]. Prior studies demonstrate that *C*. *elegans* males have a shorter lifespan than hermaphrodites when in groups [20]. However, mating and male pheromones have been shown to decrease lifespan, and males live longer than hermaphrodites when cultured individually during adulthood [20, 78, 79]. Our assays with worms isolated individually indicate that male-biases in heat and juglone resistance are not dependent on chemical or physical interactions between worms. Our assays were completed in the first few days of adulthood with worms under severe stress; these conditions likely reduce interactions between worms compared to longevity assays under standard culture conditions. The greater male stress resistance that we observed correlates with a greater intrinsic male lifespan. It is not known if sex influences stress resistance in other species including mammals that have well-established sex-biases in associated phenotypes such as degenerative disease, aging, and longevity [8–11].

Although mechanisms of resistance vary by mode and degree of stress, transcriptional regulation of genes that reduce stress (e.g., detoxification or osmolyte accumulation genes) or promote repair (e.g., heat shock protein chaperones) is central to many responses [27, 34, 37, 80]. Some canonical stress response genes were expressed greater in males than hermaphrodites under basal conditions, but these differences are difficult to interpret because of sex-specific organs and other gross anatomical differences [81]. Induction of osmotic, heat shock, and detoxification genes is well documented and understood for *C*. *elegans* hermaphrodites but not for males [27, 46, 52, 80, 82]. Our results demonstrate that males induce these stress responses by levels comparable to hermaphrodites (Fig 3) and in the same major tissues (Fig 4, S3 and S4 Figs). Therefore, we did not observe any obvious sex-specific differences in expression of core stress response genes that would explain greater stress resistance in males. It is possible that sex-specific regulation of other cytoprotective genes is responsible for differences in resistance, but global transcript comparisons between sexes have not found male enrichment for genes with obvious cytoprotective functions under basal conditions [83, 84].

### Potential post-transcriptional mechanisms of male-biased stress resistance

Given that stress response gene expression comparisons did not reveal obvious mechanisms for the male-biased stress resistance that we observed, differences in post-transcriptional mechanisms may play important roles. The activity of key cytoprotective proteins can be regulated independent of mRNA; this includes GPDH-1, which was recently shown to be regulated by protein O-GlcNAc transferase OGT-1 independently of mRNA levels [82]. Species-wide male-bias in resistance to at least two distinct types of stress, high temperature and pro-oxidant, suggest that there may be a broad mechanism not specific to any one mode of stress. Under many types of stress, global protein translation is reduced to conserve resources and reduce the burden on protein homeostasis [50, 51, 85–87]. Turn-over of damaged proteins and autophagy also promote cell homeostasis during stress [88, 89]. Future studies could compare these processes in the two sexes.

### Heat shock and juglone resistance are influenced by TRA-1

Sex determination in *C*. *elegans* is regulated by a well-characterized gene dosage-dependent signaling cascade terminating in transcriptional master regulator TRA-1 [40]. Wild type XO males and XX *tra-1* ‘pseudomales’ have low TRA-1 activity and were more resistant to heat shock and juglone than XX wild type hermaphrodites (Fig 5 and S1 Fig). These results are consistent with developmental outcomes downstream from TRA-1 that reduce heat shock resistance in hermaphrodites relative to males. TRA-1 is a transcriptional repressor and was found to bind to at least 184 genes with ChIP-seq making identification of downstream mechanisms potentially complicated [90]. However, genetic analyses have identified some key downstream regulators of sexual characteristics that could be tested for effects on stress resistance [91–94]; these include *mab-3* (Male Abnormal), *dmd-3* (Doublesex/Mab-3 Domain), *ceh-30* (*C*. *Elegans* Homeobox), *egl-1* (EGg Laying defective), *fog-1* (Feminization Of Germline), and *fog-3*; MAB-3 represses vitellogenin genes needed to provide yoke to oocytes [95–97], MAB-3 and DMD-3 control male-tail development [98], CEH-30 and EGL-1 protect male-specific neurons from undergoing apoptosis [91], and FOG-1 and FOG-3 regulate sexual characteristics of the germline [93, 94]. Mutations in these pathways feminize or masculinize these specific processes and, in future studies, can be tested for effects on stress resistance.

## Supporting information

S1 Table Sequences of primers. (XLSX)

S1 Fig Males are more resistant to osmotic stress, heat shock, and juglone than hermaphrodites as individuals. Details of individual stress survival assays. Heat shock trial 3 and juglone trial 2 are shown in Fig 2. Juglone trial 3 is shown in Fig 5B.
(PDF)

S2 Fig Basal expression of stress response genes in male and hermaphrodite N2. Relative mRNA levels of core stress response genes in young adults were measured with qRT-PCR. Values are mean plus standard errors. *N* = 7–9 replicates of 3–5 worms each. *P < 0.05, **P < 0.01, and ***P < 0.001 versus hermaphrodites.
(PDF)

S3 Fig Males induce *gpdh-1* in the same tissues as hermaphrodites. Paired bright-field and fluorescence micrographs of *gpdh-1p*::dsRed2;*myo-2p*::GFP expressing worms on agar with 51 or 250 mM NaCl. Images of the same magnification and strain were taken with the same exposure settings. Scale bars are 200 or 50 μm at low and high magnification, respectively. Images are representative of at least 10 worms.
(PDF)

S4 Fig *hsp-16*.*2p*::GFP and *gst-4p*::GFP under basal conditions. Paired bright-field and fluorescence micrographs of *hsp-16*.*2p*::GFP (A) and *gst-4p*::GFP (B) expressing worms under control conditions. Images of the same magnification and strain were taken with the same exposure settings. Scale bars are 200 or 50 μm at low and high magnification, respectively. Images are representative of at least 10 worms.
(PDF)

S5 Fig Basal expression of stress response genes in XX *tra-1* pseudomales and hermaphrodite N2. Relative mRNA levels of core stress response genes in young adults were measured with qRT-PCR. Values are mean plus standard error. *N* = 4–12 replicates of 6–12 worms each. *P < 0.05, **P < 0.01, and ***P < 0.001 versus N2 hermaphrodites.
(PDF)
