# Supplementary material for: Sexual dimorphism in Caenorhabditis elegans stress resistance
Source: PLoS One. 2022 Aug 11;17(8):e0272452. doi: 10.1371/journal.pone.0272452 (PMC9371273; doi:10.1371/journal.pone.0272452)
Supplement: S1 Fig — Details of individual stress survival assays. Heat shock trial 3 and juglone trial 2 are shown in Fig 2. Juglone trial 3 is shown in Fig 5B. (PDF) [file pone.0272452.s002.pdf]

Figure S1

Summary of single worm survival assays

Trial	Condition	no. worms	no. censure	mean (h)	std. error	P-value† vs N2 hermaphrodite	P-value† vs N2 male
Heat shock 1	N2 male	47	4*	42.13	4.05	0.0105	
	N2 hermaphrodite	48	0	28.50	2.73		0.0105
Heat shock 2	N2 male	47	10*	56.68	4.99	6.50E-06	
	N2 hermaphrodite	48	2*	25.25	3.75		6.50E-06
Heat shock 3	N2 male	47	2*	40.85	4.02	0.0055	
	N2 hermaphrodite	48	0	26.25	2.95		0.0055
Juglone 1	N2 male	47	36*	45.83	1.01	0.00E+00	
	N2 hermaphrodite	48	3**	14.63	1.26		0.00E+00
Juglone 2	N2 male	49	4*	52.78	2.68	0.00E+00	
	N2 hermaphrodite	46	0	15.91	1.01		0.00E+00
Juglone 3	*tra-1* pseudomale	48	7	52.00	2.48	0.00E+00	
	N2 hermaphrodite	47	2*	15.70	1.36		0.00E+00
Juglone 4	N2 male	35	0	63.31	2.88	0.00E+00	
	tra-1 pseudomale	46	8*	68.04	3.63	0.00E+00	0.1154
	N2 hermaphrodite	48	6**	22.05	0.75		0.00E+00

*still alive at last time interval scored, **bag of worms, †long-rank test
