# Supplementary figures and images for: Sexual dimorphism in Caenorhabditis elegans stress resistance
Source: PLoS One. 2022 Aug 11;17(8):e0272452. doi: 10.1371/journal.pone.0272452 (PMC9371273; doi:10.1371/journal.pone.0272452)

Figure S2

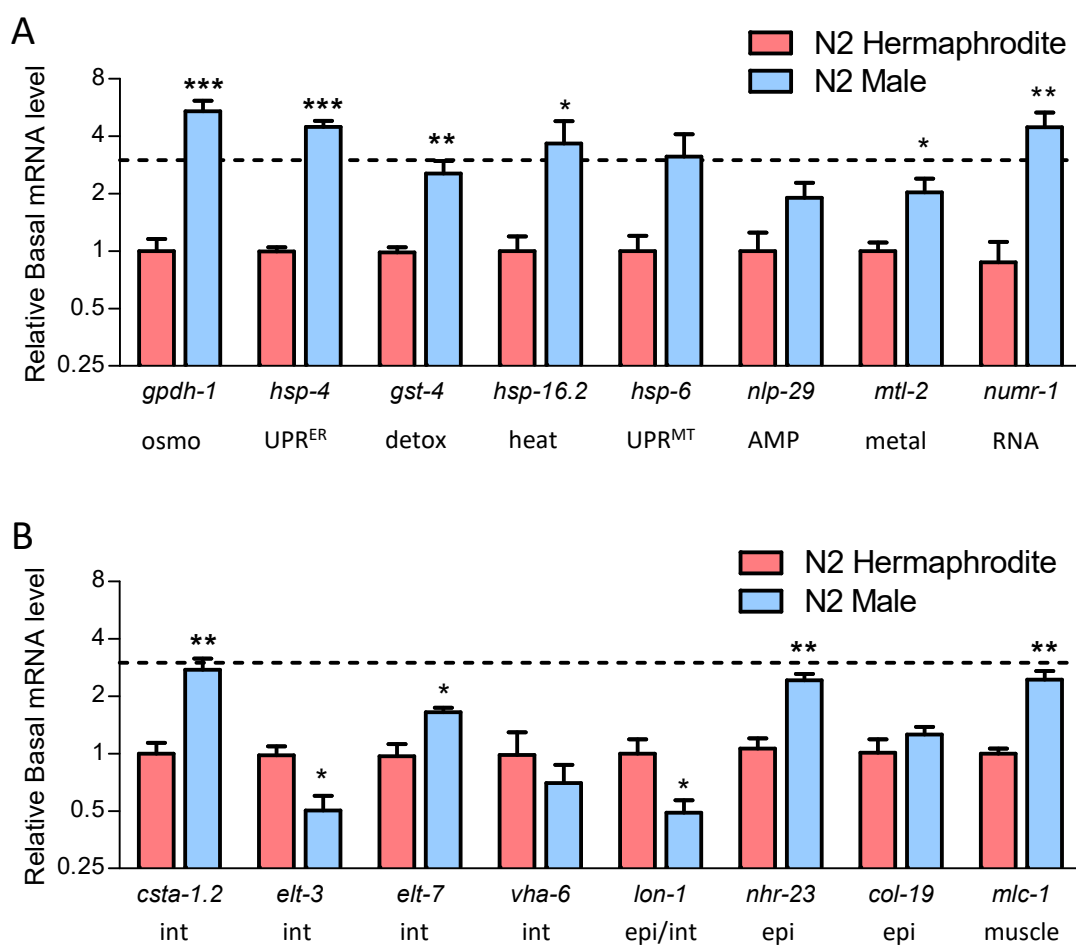

Supplement: S2 Fig — Relative mRNA levels of core stress response genes in young adults were measured with qRT-PCR. Values are mean plus standard errors. N = 7–9 replicates of 3–5 worms each. *P < 0.05, **P < 0.01, and ***P < 0.001 versus hermaphrodites. (PDF) [file pone.0272452.s003.pdf]

Figure S4

A Heat shock response - *hsp-16.2p::GFP* (green)

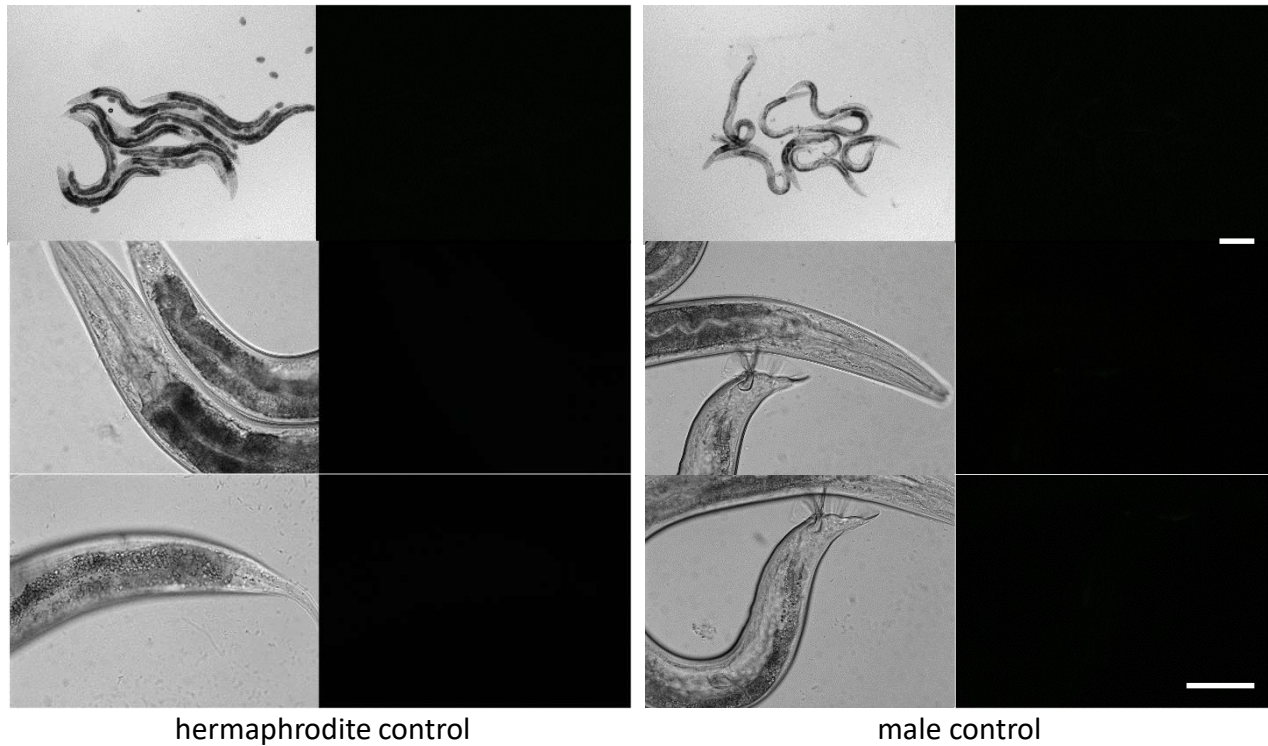

B Detoxification response - *gst-4p::GFP* (green)

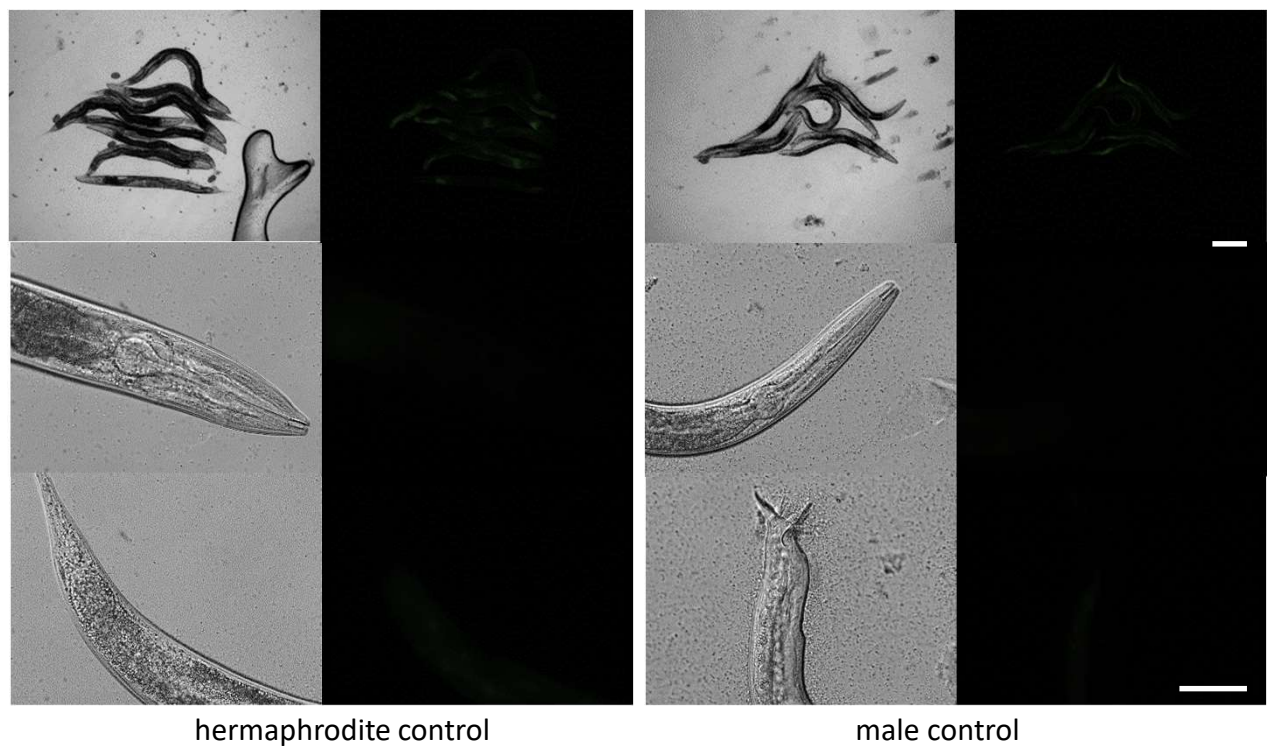

Supplement: S4 Fig — Paired bright-field and fluorescence micrographs of hsp-16.2p::GFP (A) and gst-4p::GFP (B) expressing worms under control conditions. Images of the same magnification and strain were taken with the same exposure settings. Scale bars are 200 or 50 μm at low and high magnification, respectively. Images are representative of at least 10 worms. (PDF) [file pone.0272452.s005.pdf]

Figure S5

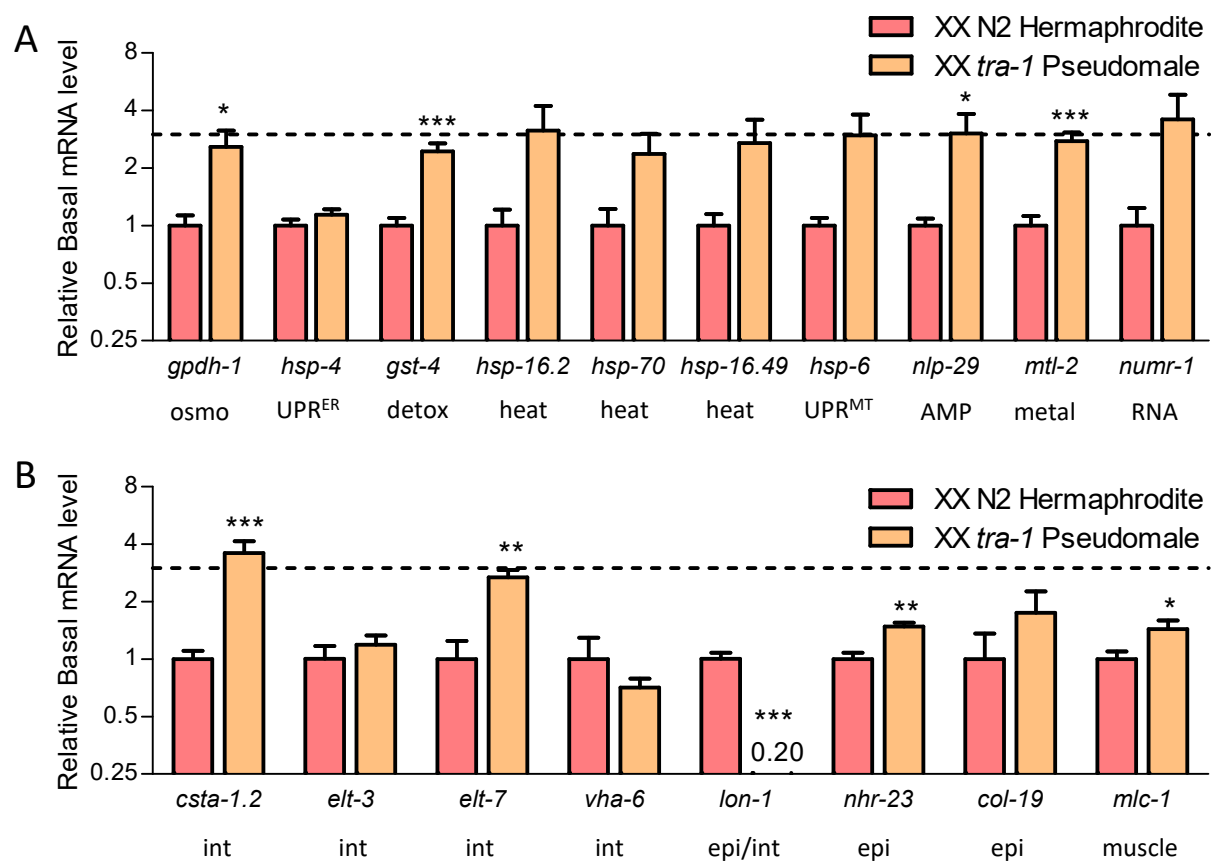

Supplement: S5 Fig — Relative mRNA levels of core stress response genes in young adults were measured with qRT-PCR. Values are mean plus standard error. N = 4–12 replicates of 6–12 worms each. *P < 0.05, **P < 0.01, and ***P < 0.001 versus N2 hermaphrodites. (PDF) [file pone.0272452.s006.pdf]
