# Supplementary material for: Sexual dimorphism in Caenorhabditis elegans stress resistance
Source: PLoS One. 2022 Aug 11;17(8):e0272452. doi: 10.1371/journal.pone.0272452 (PMC9371273; doi:10.1371/journal.pone.0272452)
Supplement: S3 Fig — Paired bright-field and fluorescence micrographs of gpdh-1p::dsRed2;myo-2p::GFP expressing worms on agar with 51 or 250 mM NaCl. Images of the same magnification and strain were taken with the same exposure settings. Scale bars are 200 or 50 μm at low and high magnification, respectively. Images are representative of at least 10 worms. (PDF) [file pone.0272452.s004.pdf]

Figure S3

A

Osmotic response - *gpdh-1p::dsRed2* (red);
myo-2p::GFP (yellow-green)

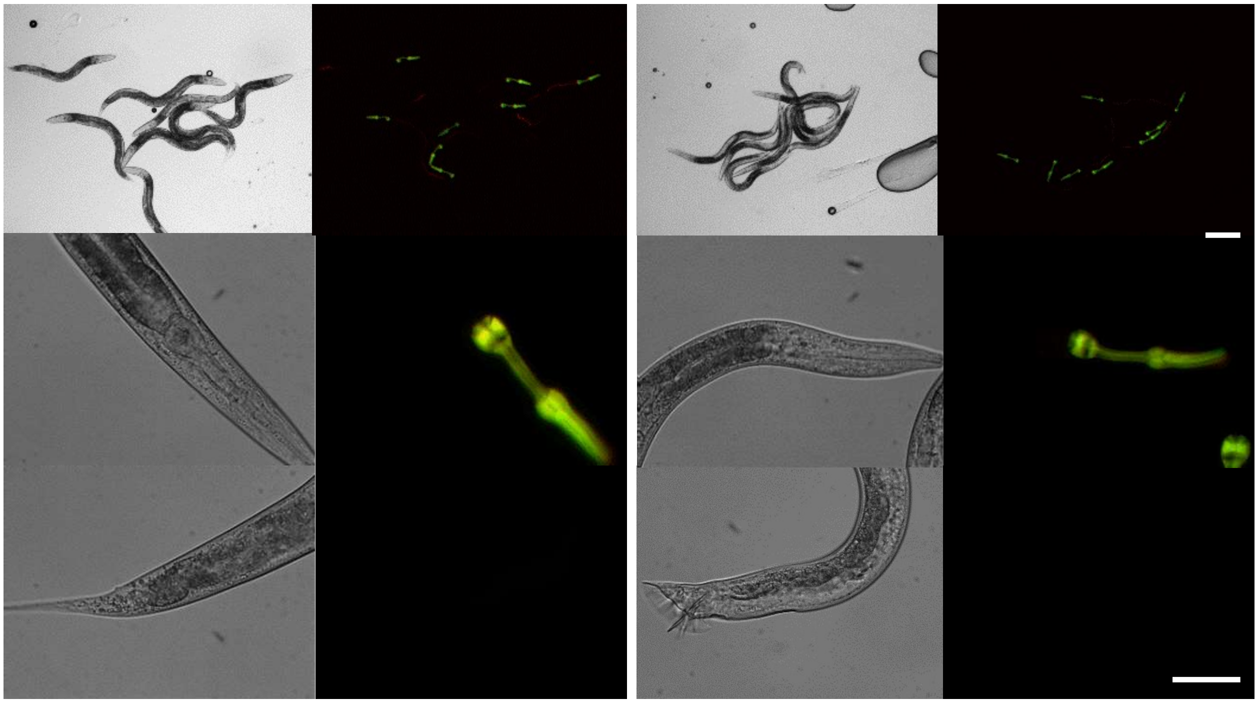

hermaphrodite 51 mM NaCl

male 51 mM NaCl

B

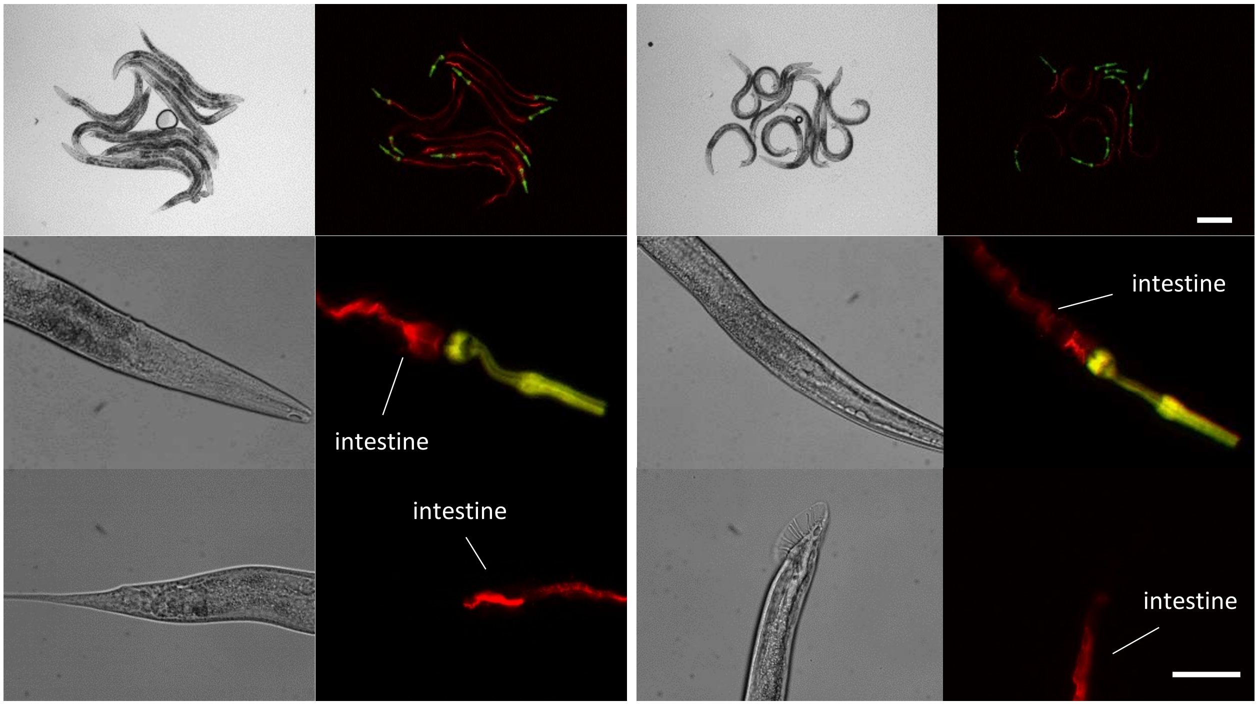

hermaphrodite 250 mM NaCl

male 250 mM NaCl
